# TranscriptClean: variant-aware correction of indels, mismatches and splice junctions in long-read transcripts

**DOI:** 10.1093/bioinformatics/bty483

**Published:** 2018-06-15

**Authors:** Dana Wyman, Ali Mortazavi

**Affiliations:** 1 Department of Developmental and Cell Biology, UC Irvine, Irvine, CA, USA; 2 Center for Complex Biological Systems, UC Irvine, Irvine, CA, USA

## Abstract

**Motivation:**

Long-read, single-molecule sequencing platforms hold great potential for isoform discovery and characterization of multi-exon transcripts. However, their high error rates are an obstacle to distinguishing novel transcript isoforms from sequencing artifacts. Therefore, we developed the package TranscriptClean to correct mismatches, microindels and noncanonical splice junctions in mapped transcripts using the reference genome while preserving known variants.

**Results:**

Our method corrects nearly all mismatches and indels present in a publically available human PacBio Iso-seq dataset, and rescues 39% of noncanonical splice junctions.

**Availability and implementation:**

All Python and R scripts used in this paper are available at https://github.com/dewyman/TranscriptClean.

## 1 Introduction

Conventional short-read RNA sequencing is widely used to quantify gene expression in a variety of applications. While cost-effective and accurate, short reads lack the ability to resolve full-length mammalian isoforms, which are commonly multiple kilobases long (Conesa, 2016). Long-read sequencing platforms such as Pacific Biosciences (PacBio) and Oxford Nanopore bypass the transcript reconstruction challenges of short reads, but have substantially higher error rates. Raw PacBio reads have a stochastic error rate of 11–15%, including single-base mismatches and microindel errors (Eid, 2009). Microindels are especially problematic during isoform mapping because they can misrepresent splice junction locations.

Circular consensus correction and read polishing steps in the PacBio ToFU analysis pipeline can substantially reduce the error rate for most transcripts once raw reads are processed (Eid, 2009; Gordon, 2015). However, this correction process is only effective when multiple sequencing passes over the same insert molecule are available, which becomes less likely as transcript length increases (Rhoads and Au, 2015).

To address this problem, various PacBio-specific tools have been developed to correct transcripts downstream of the ToFU pipeline. TAPIS, HapIso and SQANTI use a reference-guided approach to correct indels within exons (Abdel-Ghany, 2016; Mangul *et al.*, 2017; Tardaguila, 2018). HapIso distinguishes single nucleotide variants from errors in a haplotype-aware manner by phasing long reads. TAPIS and SQANTI deal with remaining errors by removing affected transcripts, the former using a splice junction quality filter, and the latter using a random forest classifier. While these methods produce cleaner PacBio datasets, none of them attempt to correct noncanonical splice junctions arising from microindel errors. Furthermore, HapIso requires multiple transcripts per gene in order for the phasing to work, which is not a given depending on sequencing depth and gene expression level.

We present TranscriptClean, a program that uses the reference genome, splice annotation and a variant file to correct mismatches, microindels and noncanonical splice junctions in PacBio transcripts while preserving known variants. Running TranscriptClean on a publicly available PacBio human transcriptome from GM12878 (Tilgner, 2014), we corrected 99% of indels, 98% of mismatches and 39% of noncanonical splice junctions present in these transcripts. This allowed us to salvage 32 536 transcripts that would have been discarded under previous workflows because of noncanonical splice junctions.

## 2 Materials and methods

### 2.1 Indel and mismatch correction

TranscriptClean processes transcripts in the SAM format, scanning each entry to look for insertions, deletions and mismatches relative to the reference genome. Indels less than or equal to the size threshold (default ≤ 5 bp) are modified to match the reference sequence. Mismatches in the transcripts are replaced with the reference base. Indel and mismatch correction can also be run in variant-aware mode to avoid removing variants of interest to the user. In this mode, mismatches and indels are changed to the reference sequence only if they do not match the position and sequence of a known variant in a user-provided VCF file. A potential downside of running mismatch correction is that it will remove novel SNPs or RNA editing events not provided in the VCF.

TranscriptClean outputs a SAM file of corrected transcripts with updated CIGAR, sequence and MD/NM fields. It also provides a fasta file of corrected sequences alongside log files tracking changes to individual errors and transcripts. The accessory script generate_report.R produces figures summarizing the TranscriptClean results, and can also be used to choose an appropriate indel size threshold for a given dataset, as the size distribution may vary across different PacBio chemistries.

### 2.2 Noncanonical splice junction correction

TranscriptClean also provides the option of correcting noncanonical splice junctions. During pre-mRNA splicing, dinucleotides at the start and end of the intron form highly conserved canonical motifs GTAG, GCAG and ATAC, with GTAG accounting for 98.9% of known human splice junctions (Dobin, 2013; Parada, 2014). Noncanonical splice junctions (NCSJs) are very rare events, which suggests that most NCSJs in long-read transcripts are likely to be sequencing errors. Typically 10–20% of PacBio transcripts contain at least one NCSJ (Tardaguila, 2018).

When a microindel error disrupts a splice boundary, the read mapping can be affected in a variety of ways. In one scenario, the entire junction is shifted upstream or downstream of its original location. In another, the error is split across the junction, resulting in a smaller indel on each side. Finally, the error may only affect one side of the junction.

To identify NCSJs, TranscriptClean checks the intron motif of each transcript splice site. Each NCSJ is compared to user-provided high-confidence splice junctions (derived from same-sample mapped short RNA-seq reads or a reference annotation) and is changed to match the known junction when the distance between the NCSJ and its nearest high-confidence junction is microindel-sized.

## 3 Results

We performed two TranscriptClean runs on CCS-processed circular consensus GM12878 PacBio transcripts from Tilgner (2014) (Table 1). In the first, we used known human splice junction annotations from GENCODE v24 and no variant file. Next we provided GM12878-specific variants and splice junctions derived from GM12878 short reads. When provided GM12878-specific references for correction, TranscriptClean corrected 99% of indels and 39% of NCSJs, rescuing 32 536 transcripts no longer considered noncanonical. 98% of mismatches were corrected, with the remaining 2% representing known NA12878 SNPs.

**Table 1.** Summary of GM12878 TranscriptClean results

	No TC	TC with GENCODE splice junctions	Corrected	TC with GM12878 Illumina SJs and variants	Corrected
Total Transcripts	568048	568048	–	568048	–
Canon. Transc.	479005	512092	–	511541	–
Noncan. Transc.	89043	55956	37%	56507	37%
Deletions	3133172	23047	99%	29883	99%
Insertions	1901787	20175	99%	21816	99%
Mismatches	14380068	0	100%	295547	98%
NCSJ	109268	66304	39%	66784	39%

A major goal of long-read isoform characterization is to provide a higher-quality reference transcriptome for short-read quantitation. If such a reference contains frequent sequencing errors, reads will not map well to it, defeating its purpose. Furthermore, downstream analysis programs commonly ignore transcripts with one or more NCSJs, effectively throwing out long-read data that could provide interesting isoform information. Repairing errors where possible allows more data to be used, particularly for longer transcripts. While the current version of variant-aware TranscriptClean does not account for the case of a sequencing error converting a real SNP to the reference base, nor the case where a real indel is disguised by one or more sequencing errors, we hope to improve correction for special cases like these in future versions and to support transcript correction of Oxford Nanopore reads.

## Funding

This work was supported by the National Human Genome Research Institute to AM [UM1 HG009443].

*Conflict of Interest*: none declared.
